# Pharyngeal electrical stimulation for postextubation dysphagia in acute stroke: a randomized controlled pilot trial

**DOI:** 10.1186/s13054-023-04665-6

**Published:** 2023-10-03

**Authors:** Sonja Suntrup-Krueger, Bendix Labeit, Thomas Marian, Jens Schröder, Inga Claus, Sigrid Ahring, Tobias Warnecke, Rainer Dziewas, Paul Muhle

**Affiliations:** 1 Department of Neurology, University Hospital Münster, Albert-Schweitzer Campus 1, A1, 48149 Münster, Germany; 2 Department of Neurology and Neurorehabilitation, Klinikum Osnabrück, Osnabrück, Germany

*Trial registration*: ClinicalTrials.gov NCT02470078 (June 12, 2015).

Dear editor

Postextubation dysphagia has been demonstrated to be the most important risk factor for extubation failure in acute stroke patients [1]. It has no proven treatment. The need for reintubation is associated with pneumonia, prolonged treatment and unfavorable outcomes.

Pharyngeal electrical stimulation (PES) is a novel neurostimulation technique that has been shown to enhance reorganization of the swallow-related motor cortex, to facilitate activation of corticobulbar pathways [2] and to increase salivary levels of substance P, a neurotransmitter involved in the control of swallowing. It has recently been proven to allow faster decannulation in severely dysphagic tracheotomized stroke patients [3, 4].

Here, we evaluated whether PES can also enhance the recovery of dysphagia early after extubation, thereby reducing dysphagia-related treatment complications.

We conducted a randomized controlled single-center pilot trial on 60 extubated acute stroke patients with severe dysphagia, defined as a score of > 4 on the validated 6-point fiberoptic endoscopic dysphagia severity scale (FEDSS) [5]. Participants were randomized within 4 h after extubation to receive either real or sham PES (10 min/day, 3 consecutive days) in addition to standard care. Stimulation was delivered via the Phagenyx system (Phagenesis Ltd, Manchester, UK). It consisted of a nasogastric feeding tube housing a pair of ring electrodes connected to a stimulation device. Catheter placement and stimulation were performed according to the manufacturer’s instructions. The primary endpoint was the need for reintubation within 120 h of extubation. Secondary endpoints were pneumonia incidence, parameters of swallowing function and feeding status, and length of stay.

Study groups did not differ in demographics, clinical condition, stroke characteristics, reason for intubation, time from stroke onset or duration of mechanical ventilation. Swallowing function was comparable at baseline. The catheter could be inserted in all patients, and there were no intervention-related complications. Four patients in the PES group and seven in the sham group did not receive the allocated intervention as per protocol because reintubation became necessary prior to completion of the three-day intervention. One patient in the sham group received three treatment sessions but needed to be reintubated before swallowing examination could be performed. All patients were included in an intention-to-treat analysis.

Reintubation became necessary in 13% (*n* = 4) of the PES group and 33% (*n* = 10) of the sham group within 120 h (*p* = 0.067). The main reason was insufficient airway protective reflexes in both groups. Extubation failure in the PES group was exclusively found within the first 30 h, i.e., prior to any PES treatment (*n* = 2) or after only one (*n* = 2) stimulation. In the sham group, reintubation events were continuously documented throughout the entire 120 h observation period (see the Kaplan‒Meier curve in Fig. 1).

**Fig. 1:**
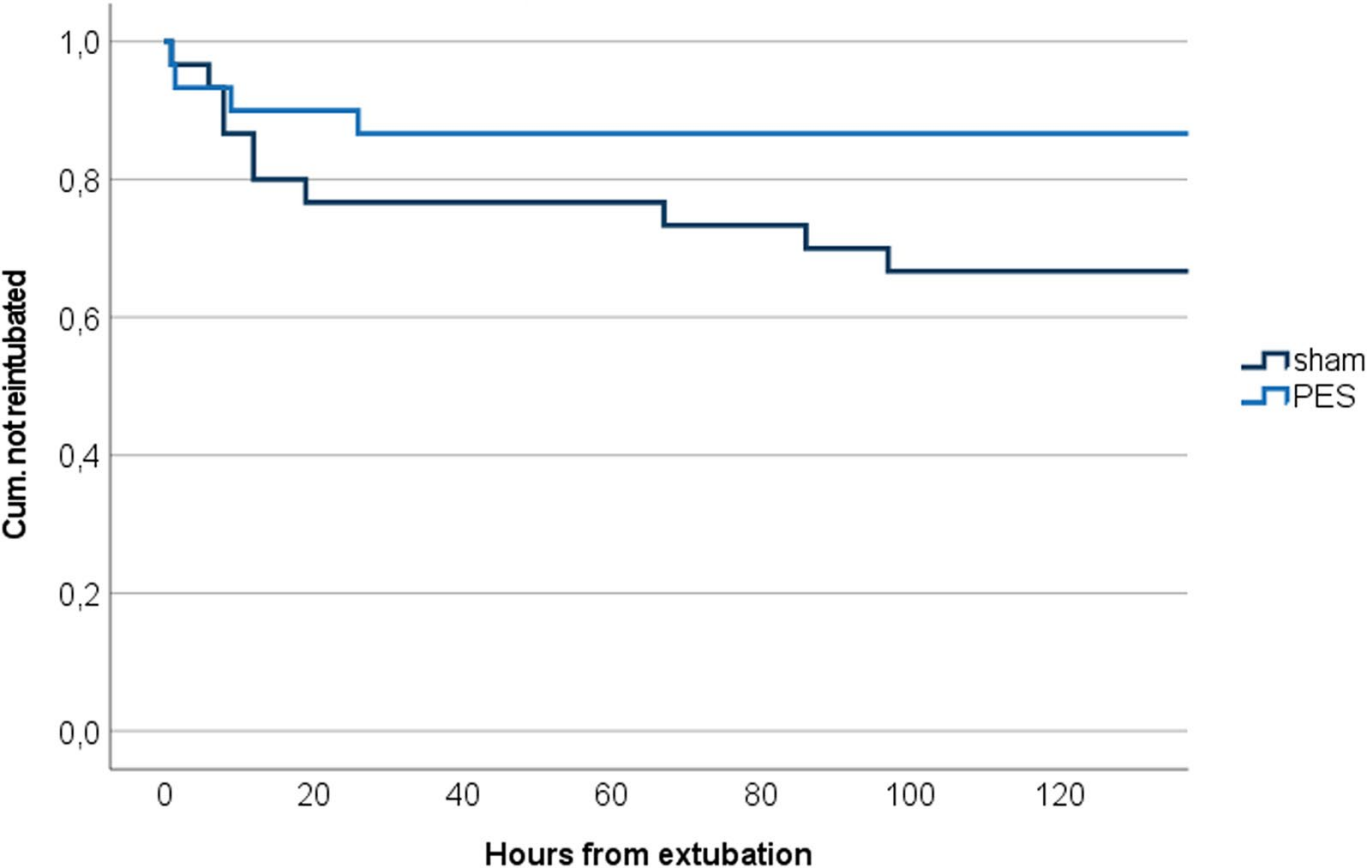
Kaplan‒Meier curves of the PES and sham treatment groups for time to reintubation from extubation (*p* = 0.083; log-rank Mantel‒Cox; *PES* Pharyngeal Electrical Stimulation)

None of the 26 PES patients who received three stimulations needed to be reintubated. The incidence of pneumonia was reduced by PES (60 vs. 83%, *p* < 0.05). This resulted in fewer days on antibiotics (4.2 vs. 8.7 p = 0.005) and a lower number of antimicrobial drugs (0.8 vs. 2.1, *p* = 0.003). Greater improvement in swallowing was observed after PES compared to sham (FEDSS 3.3 vs. 4.3 pts, *p* < 0.0005); 73% (*n* = 22) of patients in the PES group consumed a completely oral diet at discharge, i.e., no tube-feeding or parenteral intake was required, compared to 47% (*n* = 14) after sham intervention. The time until resumption of oral feeding was 4.3 vs. 10.2 days (*p* = 0.001). Following PES, 27% (*n* = 8) were feeding tube-dependent at discharge compared to 53% (*n* = 16) after sham (*p* = 0.035). The length of stay in the hospital (17.0 ± 7.9 vs. 24.3 ± 12.4 days, *p* = 0.01) was shorter in the PES group.

This is the first randomized controlled trial assessing the effect of early PES on dysphagia postextubation in ICU-treated stroke patients to prevent reintubation and further dysphagia-related complications. In this small pilot study, the primary endpoint, “need for reintubation”, scarcely missed significance. All reintubation events in patients treated with PES were observed prior to the completion of a full treatment series. This indicates that early treatment initiation and timely completion of a full treatment set is needed such that the treatment effect of PES can be accumulated and developed to clinical significance for the forestallment of emergency reintubation. Our finding is corroborated by previous PES trials that equally observed a positive association between treatment efficacy and short time to treatment [3, 4]. All secondary, nevertheless clinically relevant endpoints were significant.

In summary, PES was safe and improved severe postextubation dysphagia in recently extubated stroke patients, resulting in a reduced risk of pneumonia, less tube dependency, earlier oral nutrition, and shorter length of stay. The extubation failure rate may also be reduced after successful application of a complete treatment series. Future trials should confirm these results in a larger ICU population and potentially explore the effect of timely PES even prior to an extubation trial in intubated patients at high risk of severe dysphagia.

## Data Availability

The datasets analyzed during the current study are available from the corresponding author upon reasonable request.

## Abbreviations

FEDSS: Fiberoptic endoscopic dysphagia severity scale
PES: Pharyngeal electrical stimulation
